# COVID-19 and HIV viral load suppression in children and adolescents in Durban, South Africa

**DOI:** 10.4102/sajhivmed.v23i1.1424

**Published:** 2022-12-02

**Authors:** Asandile Mathamo, Kimesh L. Naidoo, Jienchi Dorward, Thashir Archary, Christian Bottomley, Moherndran Archary

**Affiliations:** 1 Department of Paediatrics and Child Health, Faculty of Clinical Medicine, University of KwaZulu-Natal, Durban, South Africa; 2 Department of Paediatrics, King Edward VIII Hospital, Durban, South Africa; 3 Nuffield Department of Primary Care Health Sciences, University of Oxford, Oxford, United Kingdom; 4 Centre for the AIDS Programme of Research in South Africa (CAPRISA), University of KwaZulu-Natal, Durban, South Africa; 5 Department of Engineering, University of the Witwatersrand, Johannesburg, South Africa; 6 London School of Hygiene and Tropical Medicine, London, United Kingdom

**Keywords:** paediatric HIV, viral load testing, COVID-19, children, South Africa

## Abstract

**Background:**

The coronavirus disease 2019 (COVID-19) pandemic poses challenges to paediatric and adolescent HIV treatment programme. Modelling exercises raised concerns over potential impact of disruptions.

**Objectives:**

To describe the impact of the COVID-19 pandemic on viral load (VL) testing among infants, children and adolescents on antiretroviral treatment (ART) in Durban, South Africa.

**Method:**

Routinely collected, aggregated data of monthly VL counts done on all those less than 19 years old from January 2018 to January 2022 was analysed. An interrupted time series analysis using a Prais-Winsten linear regression model, including terms for lockdowns and excess mortality determined VL trends.

**Results:**

The unadjusted mean VL was 2166 (confidence interval [CI]: 252.2) and 2016 (CI: 241.9), *P* = 0.039, and percentage VL suppression rates (72.9%, CI: 2.4% vs 73.6%, CI: 1.8%) across COVID and pre-COVID periods, showing no significant difference, *P* = 0.262. In the interrupted time series analysis, modelled monthly VL counts did not differ significantly by lockdown level (e.g., level 5 lockdown: –210.5 VLs, 95% CI: –483.0 to +62.1, *P* = 0.138) or excess mortality (–0.1, 95% CI: –6.3 to 6.1, *P* = 0.969). A significant downward trend in VL testing over time, including during the pre-COVID-19 period (–6.6 VL per month, 95% CI: –10.4 to –2.7, *P* = 0.002), was identified.

**Conclusion:**

Viral load suppression for children and adolescents were not negatively affected by COVID-19. A trend of decrease in VL testing predated COVID-19.

**What this study adds:**

Evidence presented that HIV VL testing and suppression rates in children and adolescents in a high burden setting were sustained through the COVID pandemic.

## Introduction

Antiretroviral treatment (ART) coverage for children with HIV has lagged compared to adults in sub-Saharan Africa.^1^ The Joint United Nations Programme on HIV/AIDS (UNAIDS) has set a target, framed within the ‘95-95-95 strategy’, to eliminate new HIV infections by 2030.^2^ In South Africa (SA) in 2017, 84.8% of people living with HIV (PLHIV) were aware of their status, 70.7% were on ART, and for those on ART, 87.4% had suppressed viral loads (VLs).^3^ In children and adolescents, the situation is worse, with only an estimated 54.0% of children under the age of 15 living with HIV receiving ART.^4^ The coronavirus disease 2019 (COVID-19) pandemic has disrupted healthcare provision and health-seeking behaviour and poses challenges to HIV treatment programmes.^5^

KwaZulu-Natal (KZN) has the largest HIV disease burden of all provinces in SA, and the eThekwini district has the largest child and adolescent antiretroviral programme in the province.^2^ While these programmes are making significant inroads, concerns remain regarding their efficiency and sustainability, especially with the onset of the COVID-19 pandemic.^5^ The COVID-19 pandemic has called for the extra mobilisation of existing health resources, including those allocated to HIV services.^3^ In addition, lockdown measures have resulted in delayed deliveries of ART orders and widening socio-economic inequalities exacerbating poor medical outcomes.^5,6,7,8,9^ Various modelling studies have suggested that an interruption of ART would increase mother-to-child transmission of HIV by approximately 1–6 times and potentially increase AIDS-related deaths overall in sub-Saharan Africa.^5,10^

Interventions that have been shown to enhance adherence include directly observed treatment therapy, personalised treatment plans, medication diaries, and community-based support. These are affected by COVID-19 lockdown regulations and may lead to suboptimal treatment adherence and retention to care.^9,10,11^ COVID-19 lockdowns also increase the risk of infectious diseases like tuberculosis (TB) by encouraging home quarantine and prolonged contact at the household level.^12^

The redirection of HIV healthcare funding and other resources could result in an inability to adequately support PLHIV along the continuum of care. While the most important impact of the pandemic on HIV programmes is ART interruption, the effects on HIV prevention, testing services, self-management and treatment adherence are also significant.^10,13^

The long-term implications of COVID-19 on health outcomes in infants, children and adolescents with HIV remain unknown, and there remains a lack of long-term studies on this vulnerable population in low- and middle-income countries (LMIC).

This study aims to describe the impact of the COVID-19 pandemic on VL testing among infants, children and adolescents on ART in the eThekwini district, KZN, SA.

## Methodology

This was a retrospective cohort study that reviewed the facility-level VL data of infants, children and adolescents on ART across all public sector ART clinics in the city of Durban (eThekwini district), KZN. Durban has an estimated 1.7 million PLHIV in KZN, with 48 037 children under 15 years documented to be on ART and an overall HIV prevalence of 27%.^14,15^

The study period included 26 months designated as the pre-COVID-19 period (01 January 2018 to 28 February 2020) and 23 months of the designated COVID-19 period (01 March 2019 to 31 January 2022).

South Africa implemented a level 5 lockdown (the most severe of the five designated lockdown levels) at midnight on 26 March 2020,^16,17^ and these restrictions were applied nationwide. Level 5 lockdown prohibited non-essential movement and mandated closure of non-essential businesses, schools and services. Health workers were exempt from movement restrictions, including clinical staff and data capturers. Antiretroviral treatment is provided free of charge at all clinics in eThekwini, which remained open during the entire study period. Before and throughout these lockdown periods, there were no reports of ART stock-outs in the study clinics. In addition, despite increased demands on laboratory testing for COVID-19, HIV VL testing capacity remained high and guidelines on VL testing remained unchanged throughout the study period.

Data were collected from the National Health Laboratory Service (NHLS) electronic registers and Tier.Net (three interlinked electronic registers for TB and HIV), a database for the facility-based patient management system in eThekwini district. Data on the number of VL tests done per month at each clinic are routinely recorded in Tier.Net and reported by gender and age group. During collation of the data per facility, the data were de-duplicated using a combination of first name, surname, date of birth and gender. We collected data from children and adolescents up to and including 19 years of age for the study period. We used predefined periods related to the national lockdowns and COVID-19 waves determined by the National Institute for Communicable Diseases Unit (NICD). Anonymised facility-level data were aggregated into monthly counts of numbers of VL tests done in specific age groups: 0–12 months, 12–60 months, 60 months – 15 years and 15–19 years. We categorised the VL results into three groups: under 10 000 copies/mL, between 10 000 and 100 000 copies/mL and over 100 000 copies/mL. We used excess mortality data for eThekwini from the South African Medical Research Council report on weekly deaths in South Africa.^18^

### Data analysis and interpretation

We used descriptive statistics to summarise demographic data and numbers of VLs taken per month during the study period. We also calculated the mean VL tests per month before and during COVID-19. Because this does not consider pre-existing trends in VLs, we conducted interrupted time series analyses by fitting a Prais-Winsten linear regression model to account for autocorrelation. The model included a time variable, a continuous variable for excess mortality in eThekwini, a dummy variable for each SA lockdown level 1 to 5, and a dummy variable for December when clinical activities are generally much reduced due to SA national holidays. This approach considers pre-lockdown trends and allows estimation of the effect of COVID-19 restrictions through lockdowns and the impact of COVID-19 waves on healthcare. Because different severe acute respiratory syndrome coronavirus 2 (SARS-CoV-2) variants caused different levels of morbidity and mortality, we used excess mortality as a proxy for the impact of COVID-19 epidemic waves on the healthcare system. We conducted a sensitivity analysis with a negative lag of one month for excess mortality to account for the delays between SARS-CoV-2 infection, illness, hospitalisation and death. We used a separate dummy variable for each lockdown level to determine whether only a certain lockdown level affected VL numbers. We also built separate models for each age group. We analysed data using R 4.2 (R Foundation for Statistical Computing, Vienna, Austria).

### Ethical considerations

This work was approved by the University of KwaZulu-Natal Biomedical Research Ethics Committee (BREC/00003541/2021), the KwaZulu-Natal Department of Health’s Provincial Health Research Ethics Committee (KZ_202111_016), the eThekwini Municipality Health Unit, and the eThekwini District Health Department, with a waiver for informed consent for analysis of anonymised, routinely collected data.

## Results

During the study period that extended from 01 January 2018 to 31 January 2022, a total of 102 624 VL tests were done on all patients 19 years and younger across all clinics in the eThekwini district. Of these, 2293 (2%) were from infants under one year of age, 8089 (8%) from children between one and five years, 52 704 (51%) from children between five and 15 years and 39 538 (39%) in those aged 15 to 19 years.

Across all age groups, the mean number of VLs done per month was 2095 (standard deviation [s.d.]: 256, range: 1472–2652 samples). Table 1 compares the unadjusted mean number of VLs done between the pre-COVID-19 and COVID-19 periods. While the mean was significantly less in the COVID-19 period, there was no significant difference in the VL suppression between these periods.

**TABLE 1:** Comparison of viral loads done and percentage of patients virologically suppressed over the study period.

Period	COVID-19 (*N* = 23)					Pre-COVID-19 (*N* = 26)					*P*-value*
	*N*	%	Mean	s.d.	Minimum – maximum	*N*	%	Mean	s.d.	Minimum – maximum	
**Number of VL tests**											
All ages < 19 years	-	-	2016.3	241.9	1568–2652	-	-	2166.2	252.3	1568–2652	0.004
**Number of viral load tests per age categories**											
0–12 months	50.3	11.1	-	-	-	43.7	11.5	-	-	-	0.043
12–60 months	144.3	21.0	-	-	-	183.5	30.9	-	-	-	< 0.001
60 months–15 years	1002.9	145.8	-	-	-	1139.9	140.8	-	-	-	0.002
15–19 years	818.7	83.6	-	-	-	796.4	98.5	-	-	-	0.400
**Patients virologically suppressed**											
All ages < 19 years	-	-	72.9	2.4	67.3–76.5	-	-	73.6	1.8	69.8–77.3	0.026

Note: *N* = number of months.

s.d., standard deviation.

* , *P*-value from a two-sample *t*-test.

Of all the VLs done in the study period, 73% were below 1000 copies/mL, indicating viral suppression. The percentage of patients virologically suppressed by age group was 66%, 63%, 76% and 73% for children below 1 year, 1–5 years, 5–15 years and 15–19 years. There was no significant difference in the percentage of virologically suppressed patients when we compared the pre-COVID-19 to the COVID-19 periods.

### Interrupted time series analysis

**FIGURE 1:**
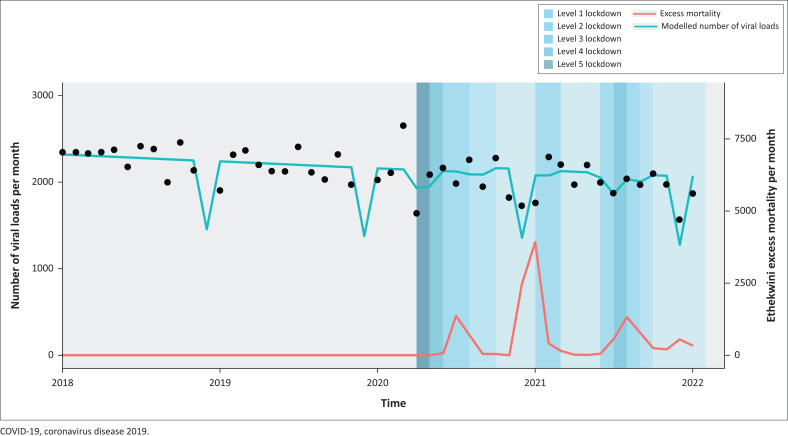
Trends in monthly viral loads among under-19-year-olds before and during COVID-19, and relationships with lockdown levels and excess mortality in eThekwini, South Africa.

In the linear regression model of numbers of monthly VLs taken among all patients 19 years old and younger that considers pre-COVID-19 trends, there was strong evidence that fewer VLs were done in December than in other months (partly due to fewer clinic days available in this annual holiday month compared with other months) (Table 2 and Figure 1).

**TABLE 2:** Linear regression model of trends in monthly viral loads taken among all under-19s in eThekwini from January 2018 to January 2022.

Variable evaluated	Change in monthly viral loads done	95% confidence interval	*P*-value
December 2020 & 2021	−785.9	−942.5 to -629.2	< 0.001
**Impact of lockdown levels**			
Lockdown level 5	−210.5	−483.0 to 62.1	0.138
Lockdown level 4	−182.6	−401.2 to 36.0	0.109
Lockdown level 3	1.7	−167.4 to 170.1	0.985
Lockdown level 2	−24.1	−184.9 to 136.7	0.770
Lockdown level 1	58.1	−70.8 to 187.0	0.382
Time (per month)	−6.6	−10.4 to -2.7	0.002
**Impact of excess mortality data in South Africa for COVID-19 period**			
Excess mortality†	−0.1	−6.3 to 6.1	0.969

† , Per 100 excess deaths.

Although there was some suggestion that the number of VLs done reduced during level 5 lockdown and level 4 lockdown, neither of these effects was statistically significant. Of note, there was a large increase in VLs in the month before the level 5 lockdown, which may have offset the impact of the level 5 lockdown.

Overall, there was evidence of a gradual decrease in the number of VLs taken over time (a decrease of six VLs per month). This trend was apparent in both the pre-COVID-19 and the COVID-19 periods.

There was no evidence of an impact of COVID-19 variants (using excess mortality as a proxy for COVID-19 disruption to healthcare services) during various waves (Table 2 and Figure 1), including in sensitivity analysis using a 1-month lag for excess mortality that considers the delay between epidemic peaks in SARS-CoV-2 infections and subsequent deaths.

**FIGURE 2:**
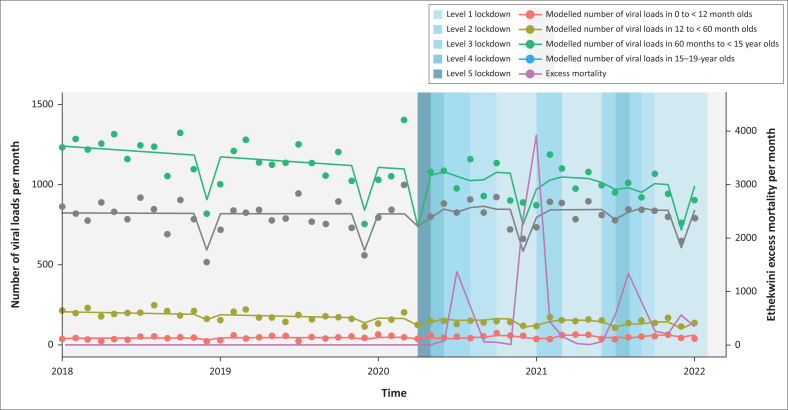
Interrupted time series analysis by age group.

In analyses stratified by age (Table 3 and Figure 2), there was evidence that VLs decreased in December 2020 and December 2021 for all age groups, except those under 1 year old. Viral load numbers decreased with time in 1–4-year-olds and 5–15-year-olds. There was also evidence that the level 5 lockdown led to a decrease in VLs taken among 5–15-year-olds.

**TABLE 3:** Linear regression analyses of monthly viral loads stratified by age.

Age groups	0 – 12 months			12 – 60 months			60 months – 15 years			15 – 19 years		
	VL change	95% CI	*P*	VL change	95% CI	*P*	VL change	95% CI	*P*	VL change	95% CI	*P*
Time (months)	0.02	−0.2 to 0.7	0.340	−1.6	−2.5 to -0.7	0.001	−5.0	−7.2 to 2.9	< 0.001	0.2	−1.5 to 1.8	0.841
December 2020 & 2021	−10.07	−21.6 to 0.3	0.064	−30.2	−52.5 to -7.9	0.011	−422.0	−511.0 to -333.0	< 0.001	−300.2	−366.2 to 234.3	< 0.001
Excess mortality (per 100 excess deaths)	0.00	−0.5 to 0.5	0.992	−0.8	−1.8 to 0.3	0.162	0.9	−2.6 to 4.4	0.623	−0.6	−3.3 to 2.0	0.632
Lockdown level 5	−11.48	−32.8 to 9.9	0.298	−42.9	−85.7 to 0.0	0.057	−198.4	−353.2 to -43.7	0.016	4.4	−110.4 to 119.2	0.940
Lockdown level 4	−3.08	−21.7 to 15.0	0.721	−20.3	−56.6 to 16.0	0.279	−92.2	−216.5 to 32.0	0.154	−61.4	−153.2 to 30.4	0.197
Lockdown level 3	−7.07	−23.8 to 8.4	0.352	3.2	−28.3 to 34.7	0.843	−25.1	−121.0 to 70.9	0.612	30.3	−41.1 to 101.7	0.410
Lockdown level 2	−1.08	−20.0 to 40.1	0.838	3.5	−30.3 to 37.2	0.841	−60.3	−151.3 to 30.7	0.201	41.1	−27.6 to 109.8	0.248
Lockdown level 1	9.03	−6.2 to 24.8	0.245	10.0	−20.0 to 40.1	0.516	8.5	−64.4 to 81.3	0.821	21.0	−34.3 to 76.4	0.461

CI, confidence interval; VL, viral load.

## Discussion

This study reports on a large cohort of children and adolescents in HIV care in KZN spanning four years. We found that while the COVID-19 pandemic had no marked impact on VL testing and suppression rates, there were significant decreases in VL testing rates before the COVID-19 pandemic, which persisted during COVID-19.

Several modelling studies and early reviews from LMIC have raised concerns about the sustainability of ART programmes within the fragile healthcare systems in sub-Saharan Africa following the COVID-19 pandemic. Studies that reviewed care among children and adolescents during the COVID-19 pandemic documented decreases in general paediatric admissions and general outpatient and emergency visits across the world, including SA.^1,19,20,21^ In this study, while VL testing decreased between the pre-COVID-19 and COVID-19 periods, this was largely due to continuation of a pre-existing trend of decreased VLs before the COVID-19 pandemic, as demonstrated in the linear regression comparison models. There was also some evidence that level 5 lockdown impacted significantly on VL numbers among 5–14-year-olds who are mostly schoolgoing children, but the impact of lockdown measures was not significant in other groups or at other lockdown levels. Viral load testing can be a proxy marker of both retention in care and the delivery of appropriate monitoring while in care. These findings correlate with other studies that indicated the general preservation of ART clinic visits even during the COVID-19 pandemic.^1,6,12,22^ We also found VL suppression rates of more than 70% in this study population over both study periods, similar to other sub-Saharan Africa studies pre-COVID-19.^23,24^ Viral load suppressions rates in this study were maintained throughout almost two years of the COVID-19 period and a similar period preceding, indicating infants, children and adolescents have maintained adherence to ART regimens. Both these findings suggest that the ART programmes in the district have the robustness to continue to provide similar care to clients already in the programme and on ART despite the pandemic-related disruptions experienced.

Previous studies from LMICs have rarely evaluated ART treatment programmes longitudinally over the different COVID-19 waves and various national lockdown levels imposed by a national government. In this study, using excess mortality as a proxy, the linear regressions model indicated that ART programmes were sustained during all varying levels of disruption experienced during varying COVID-19 waves and various lockdown levels. Pre-lockdown stocking up, multi-month prescribing and differentiated ART delivery platforms have been documented as possible reasons for the sustained care seen specifically in the ART programmes.^22,25,26^ This maintenance of care contrasted sharply with decreases noted over the same time periods with general paediatric hospitalisation rates and outpatient visits. These findings add to the limited data on the impact of the COVID-19 pandemic on paediatric ART programmes that remained robust for those already on treatment. Lessons learned from these programmes can be used to inform other care platforms delivering chronic care to patients.^21,27^

Of significance, this study identified a concerning trend of decreasing VL testing, especially in children between four and 15 years of age. The decrease preceded the COVID-19 pandemic and suggests fundamental shifts in ART care may be at play. The VL test guidelines remained the same during the study period, recommending annual VL testing for patients in chronic ART care. In 2019, the South African National Department of Health (NDoH) introduced Dolutegravir to the programme for children and adolescents over 20 kg with a suppressed VL. We would have expected an increase in VL testing in 2019, which was not seen in this study. Apart from guideline changes, service delivery changes may impact the SA ART programme, including the large KZN programme catering to children and adolescents. These include active encouragement of down-referral of children from hospital-centric clinics to primary healthcare clinics and amalgamation of HIV care into general outpatient services in larger facilities. Many of these down-referrals result in a move away from previous child-friendly clinical, social and peer group support, which is much needed.^9,11^ In addition, non-governmental support for many of these clinics, including additional staffing, has diminished since 2018, mainly due to funding challenges. The COVID-19 pandemic has exacerbated this.^26^ Increasing task shifting to clinics, with existing staff shortages, may also be diluting support and specialised care afforded to families deemed at risk of complying with the strict compliance requirements for the SA ART programme. The COVID-19 pandemic has exacerbated the socio-economic strife for many such at-risk families, which may limit their ability further. The trend in VL testing rates away from rather than towards achieving the UNAIDS goal of 95% of children on ART having a suppressed VL requires a more urgent in-depth understanding.

### Limitations

This study focuses on one district in one province of SA and may not reflect specific challenges or strengths in programmes across the country. However, KZN has the largest proportion of patients in HIV care in the country, and the eThekwini district is home to almost 50% of the province. This study did not utilise patient-specific data but rather aggregated facility-level data; therefore, we cannot directly translate the number of VLs performed to the number of patients in care. Of note, this study did not evaluate HIV testing and ART initiation rates which have been found to have decreased with the COVID-19 pandemic in other studies.

## Conclusion

Despite changes in both lockdown restrictions and the burden of disease, the number of both VL tests done and the percentage of unsuppressed VLs were not markedly impacted by the COVID-19 pandemic. Paediatric VL testing across the eThekwini district, KZN, remained largely robust through the COVID-19 pandemic negating the predicted negative modelled scenarios. However, a long-term trend of slow reduction in the number of VLs done preceding the COVID-19 pandemic requires further evaluation. It may be due to changes in service delivery to children on ART in the district.
